# Genome-scale metabolic modeling reveals increased reliance on valine catabolism in clinical isolates of *Klebsiella pneumoniae*

**DOI:** 10.1038/s41540-022-00252-7

**Published:** 2022-10-28

**Authors:** Matthew L. Jenior, Mary E. Dickenson, Jason A. Papin

**Affiliations:** 1 Department of Biomedical Engineering, University of Virginia, Charlottesville, VA USA; 2 Department of Medicine, Division of Infectious Diseases & International Health, University of Virginia, Charlottesville, VA USA; 3 Department of Biochemistry & Molecular Genetics, University of Virginia, Charlottesville, VA USA

**Keywords:** Computer modelling, Systems analysis, Pathogenesis

## Abstract

Infections due to carbapenem-resistant Enterobacteriaceae have recently emerged as one of the most urgent threats to hospitalized patients within the United States and Europe. By far the most common etiological agent of these infections is *Klebsiella pneumoniae*, frequently manifesting in hospital-acquired pneumonia with a mortality rate of ~50% even with antimicrobial intervention. We performed transcriptomic analysis of data collected previously from in vitro characterization of both laboratory and clinical isolates which revealed shifts in expression of multiple master metabolic regulators across isolate types. Metabolism has been previously shown to be an effective target for antibacterial therapy, and genome-scale metabolic network reconstructions (GENREs) have provided a powerful means to accelerate identification of potential targets in silico. Combining these techniques with the transcriptome meta-analysis, we generated context-specific models of metabolism utilizing a well-curated GENRE of *K. pneumoniae* (iYL1228) to identify novel therapeutic targets. Functional metabolic analyses revealed that both composition and metabolic activity of clinical isolate-associated context-specific models significantly differs from laboratory isolate-associated models of the bacterium. Additionally, we identified increased catabolism of L-valine in clinical isolate-specific growth simulations. These findings warrant future studies for potential efficacy of valine transaminase inhibition as a target against *K. pneumoniae* infection.

## Background

Carbapenem-resistant Enterobacteriaceae (CRE) have emerged as a growing and urgent issue in healthcare facilities around the world, posing a significant threat to public health. Carbapenem antibiotics, currently considered to be the most potent and highly effective class of antimicrobial agents, are often considered a last-resort, reserved specifically for the treatment of severe multidrug-resistant (MDR) bacterial infections^1–3^. This recent surge in CRE-associated infections has been driven primarily by the emergence and dissemination of carbapenemases, a specific type of β-lactamase that has the ability to hydrolyze carbapenems, rendering even carbapenem-class antibiotics ineffective^2^. A large proportion of these CRE-related infections are due to the Gram-negative bacterium *Klebsiella pneumoniae*^2,3^, with over 50% of *K. pneumoniae* infections now being resistant to carbapenems in parts of the Eastern Mediterranean and Europe^3^. As *K. pneumoniae* has been rapidly acquiring antibiotic resistance and rendering almost all available treatments ineffective, the discovery of new treatment strategies for this bacterial pathogen are critical^1,3^.

One strategy that has emerged recently is the targeting of elements of virulence or core metabolism that may be too costly for the organism to accumulate mutations in or diminish the ability to manifest disease^4^. By identifying those characteristics lost during evolution toward sustained laboratory culture, while remaining conserved across infections, it becomes possible to gain insight into important phenotypes that contribute to successful infection. Furthermore, it has been shown that clinical and laboratory isolates of other bacterial pathogens may also be easily differentiated by distinct metabolic capacities^5^. Employing this approach for *K. pneumoniae*, we may highlight “core” metabolic pathways in clinical isolates that may present ideal therapeutic target candidates. Consistent with this strategy, certain elements of metabolism have already been successfully identified as drug targets in bacterial pathogens including other Enterobacteriaceae^6–9^.

Changes in bacterial transcription have been used to assess differences in active metabolism with higher resolution than metabolomics screens, as shifts can be traced to specific pathways and gene products^10^. While RNA-seq has become a relatively standard method for characterizing transcription, technical variability, small sample sizes, and sample heterogeneity still exist and may influence study-specific results^11,12^. Additional differences in data processing criteria also introduce variability into downstream interpretations^13,14^. To account for these factors, meta-analyses of transcriptomic datasets across multiple studies can be performed using a unified curation and analysis pipeline. As such, we assembled 56 publicly available transcriptomes of *K. pneumoniae* isolates from both the laboratory and clinical profiles during growth in similar media conditions at multiple institutions^15–18^. Overall, certain transcriptional patterns varied consistently between clinical and laboratory isolates, and differential expression analysis revealed increased transcription of aminoglycoside degradation and key regulators for histidine utilization among clinical isolates.

To further explore possible targets within infection-associated metabolic pathways, we integrated our transcriptomic meta-analysis with a previously published genome-scale metabolic network reconstruction (GENRE) of *K. pneumoniae* (iYL1228)^19^. GENREs are computational formalisms of the biochemical reactions encoded for in an organism’s genome^20,21^. Previously, GENRE-based growth simulations in other pathogens have successfully highlighted novel enzyme targets which were subsequently validated in the laboratory, effectively accelerating research efforts^8,20,21^. Additionally, GENREs can also be utilized to provide improved context for omics data as the network architecture can reveal additive effects of small changes in activity across interconnected pathways^22^. These network-based analyses enable greater insight into metabolic patterns that correspond with growth under specific conditions, such as during active infection^20,21^. We continued the transcriptomic meta-analysis through integration with metabolic network-based investigation which allowed us to discern novel conserved components of *K. pneumoniae*’s metabolic strategy specific to active infection. Most prominent among these predictions was significantly elevated uptake and utilization of environmental L-valine through the increased activity of an Enterobacteriaceae-specific valine transaminase. This elevated uptake of L-valine was observed across >89% of clinical isolate context-specific models, while nearly entirely absent from models of laboratory strains, supporting the hypothesis of increased importance for survival in vivo. These results also agreed with previous findings that macrophages respond to high concentrations of exogenous valine in order to upregulate phagocytosis and the killing of *K. pneumoniae* during infection^23^. Our study highlights the utility of well-curated GENREs integrated with transcriptomic data to accelerate molecular target identification.

## Results

### Transcriptomic data collection

We performed an extensive search of publicly available RNA-Seq datasets on the NCBI Sequence Read Archive^24^ characterizing either laboratory or clinical isolates of *K. pneumoniae*. Datasets were considered for meta-analysis if isolates were grown to exponential phase in vitro using LB growth medium at 37 °C, at which point transcriptomic samples were collected and sequenced. This selected standard for transcriptomic samples was still anticipated to be reflective of emergent properties within both laboratory strains and clinical isolates^25^. Strains that undergo serial passaging, including strains that originated from the clinic, are understood to have many different characteristics when compared to strains which were more recently isolated from a clinical setting^26,27^. Therefore, the strains represented throughout this study were classified as laboratory strains if they had undergone serial passaging (defined within this study as having undergone greater than four passages^26–29^), whereas strains were classified as clinical isolates if they were recently isolated (having undergone a maximum of four passages) from a clinical setting. As properties characteristic of laboratory strains versus clinical isolates are typically understood to be developed over an extended period of time^30^. These criteria resulted in 56 total RNA-Seq datasets across four distinct studies; of these datasets, 17 represented laboratory strains^16,17^ and 39 represented clinical isolates^15,18^ (Supplementary Table 1). The combination of studies from a variety of independent groups also helps to minimize concerns due to strain or experimental variation inherent to each individual study^31^. Further, the simple categorization of datasets, being laboratory or clinical, selected for use here is both highly clinically relevant and results in a much greater ability to power the claims being made through this study.

### Transcriptomic meta-analysis reveals differential expression in metabolic regulators and antibiotic resistance

**Fig. 1:**
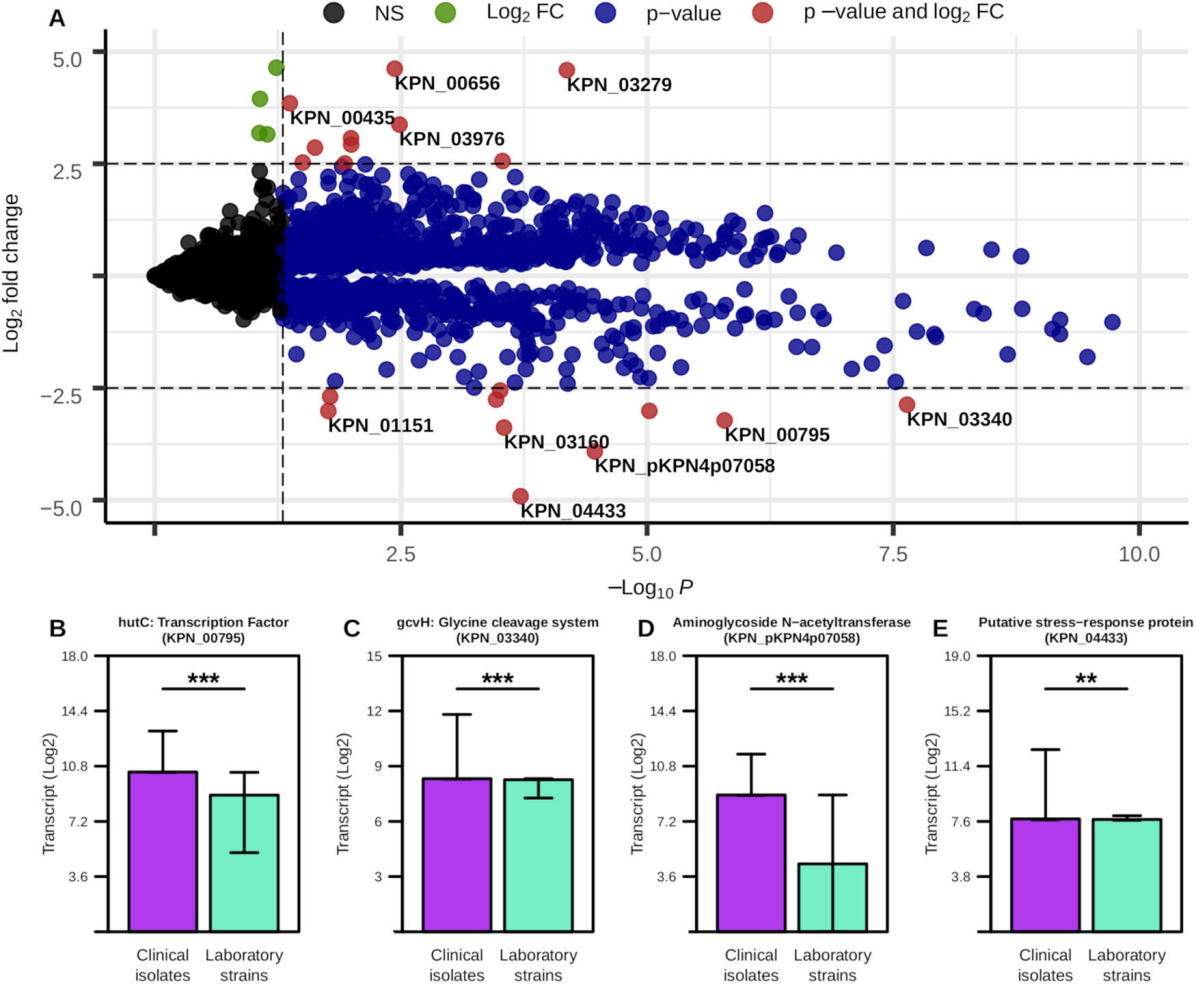
Transcriptional differences between laboratory and clinical isolates of *K. pneumoniae*. **A** Differential expression analysis with Log_2_ fold change cutoff = 2.5, *p*-value cutoff = 0.05. Genes with highest degree of difference are labeled. Each point is an individual gene and the color of the point corresponds to whether or not the Log_2_ fold change is not significant (black, NS), has a Log_2_ fold change > 2.5 (green, Log_2_ FC), has a *p*-value < 0.05 (blue, *p*-value), or a Log2 fold change > 2.5 and a *p*-value < 0.05 (red, *p*-value and log_2_ FC). **B**–**E** Median and interquartile ranges for select genes based on previous analysis. Significant differences determined by Wilcoxon rank-sum test with Benjamini–Hochberg correction (***p-value ≤ 0.001, **p-value ≤ 0.01).

To first characterize overall transcriptional dissimilarities between isolate types, we performed a differential expression analysis, comparing the relative transcriptional expression of each gene in laboratory versus clinical strains, thresholding with an adjusted *p*-value cutoff of 0.05 and a log_2_ fold change of 2.5 (Fig. 1A and Supplementary Table 2). This analysis identified a total of 19 genes as differentially expressed, 10 that are more associated with clinical isolates and 9 that are more associated with laboratory strains. Among genes with the highest degree of change were a subset of amino acid metabolism regulators, including the *hutC* transcription factor gene (Fig. 1B) and the *gcvH* glycine cleavage system (Fig. 1C), that were both significantly increased in clinical isolates. Importantly, each of these discussed transcriptional differences were statistically significant, albeit some have a low difference in absolute value of the change. Specifically, *hutC* encodes for an established regulator of histidine utilization which has been shown to be a critical colonization factor among certain strains of *K. pneumoniae*^32^. Additionally, transcript for aminoglycoside N-acetyltransferase is significantly overrepresented in clinical isolates (Fig. 1D) and encodes for an enzyme that directly mediates the breakdown of aminoglycoside antibiotics. Additionally, a putative stress response protein was expressed more highly in clinical isolates (Fig. 1E), and has also been previously associated with increased antibiotic resistance^33^. Alternatively, *mrkA*, a type-3 fimbriae subunit gene, has been shown to facilitate biofilm formation^34^ and whose expression was significantly more associated with laboratory isolates (Supplementary Fig. 1A). Furthermore, genes for multiple ribosomal subunits were highly expressed in laboratory strains (Supplementary Fig. 1B, C). This result suggests that the laboratory strains studied here may have evolved towards optimization for faster growth in culture medium^35^. Cumulatively, these results support the observation that overall transcriptional activity strongly differed between laboratory and clinical isolates in a manner that may impact infection outcomes, and underscored the point that key metabolic shifts may play a role in these differences.

### Leveraging transcriptomics and GENREs to generate context-specific models of *K. pneumoniae*

**Fig. 2:**
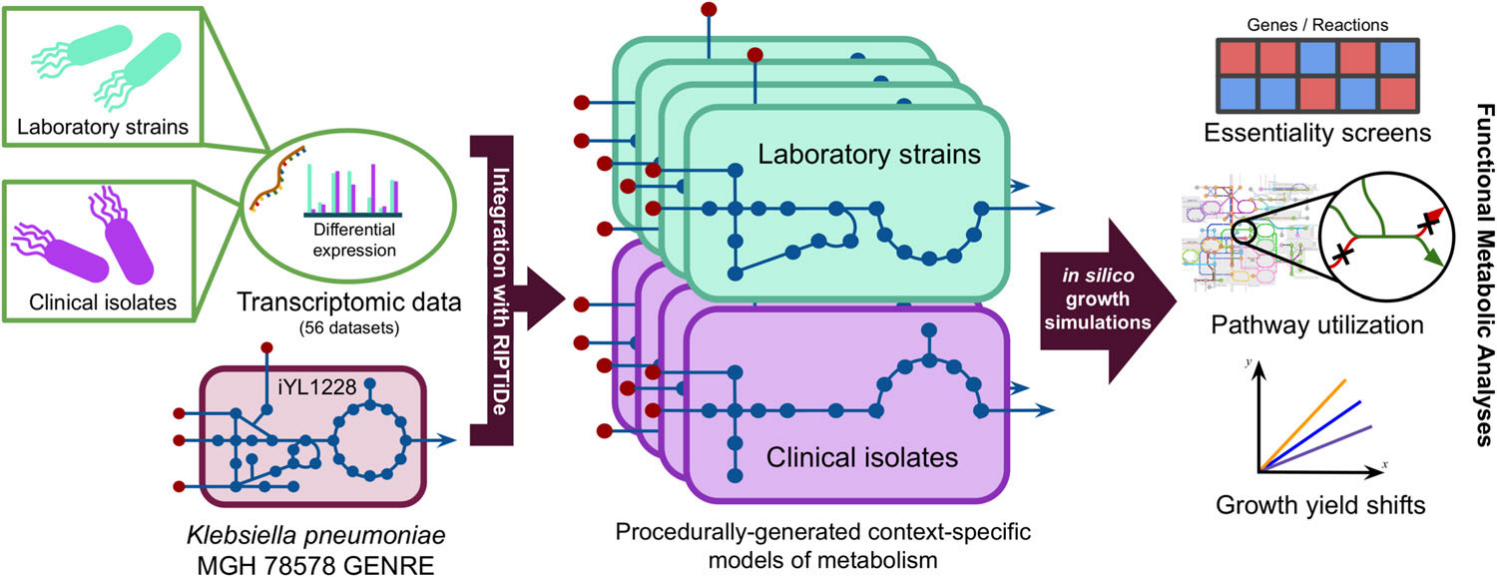
General procedure for generating context-specific models of metabolism from transcriptomic data. All 56 datasets from the transcriptome meta-analysis were used to generate distinct context-specific models of *K. pneumoniae* metabolism.

Previous studies have shown that GENREs are powerful platforms for transcriptomic data integration, allowing for the capture of greater context surrounding metabolic shifts between varying conditions^22^. We therefore generated context-specific models of metabolism representing either clinical or laboratory *K. pneumoniae* isolates utilizing a recently published method for transcriptome data integration^36^, alongside a well-curated *K. pneumoniae* GENRE (iYL1228)^37^. Briefly, the transcriptomic data integration method identifies the most cost-effective usage of metabolism to achieve growth that best reflects the cell’s investments into transcription and further prunes inactive reactions^36^. Using this approach, we generated unique isolate-type-specific models of *K. pneumoniae* metabolism in rich medium for each of the 56 collected transcriptomes, and assessed the emergent differences in active metabolism (Fig. 2). The resultant models of context-specific metabolism contained a median of 298 and 302 reactions in laboratory or clinical isolate models, respectively, from the total 2262 reactions in the uncontextualized iYL1228 (Supplementary Table 3). Interestingly, models derived from clinical isolate transcriptomic data were consistently larger than those from laboratory strains, reflecting possible loss of unnecessary metabolite biosynthesis during evolution toward growth in rich in vitro culture medium. This data-driven minimization of the possible metabolic solution space more readily reveals critical elements of context-specific metabolism of the organism otherwise not detectable from strictly analyzing the transcriptomic data, and allows for a variety of downstream growth simulations.

### Network topological analysis and essentiality screens highlight valine catabolism as differentially critical in clinical isolates

**Fig. 3:**
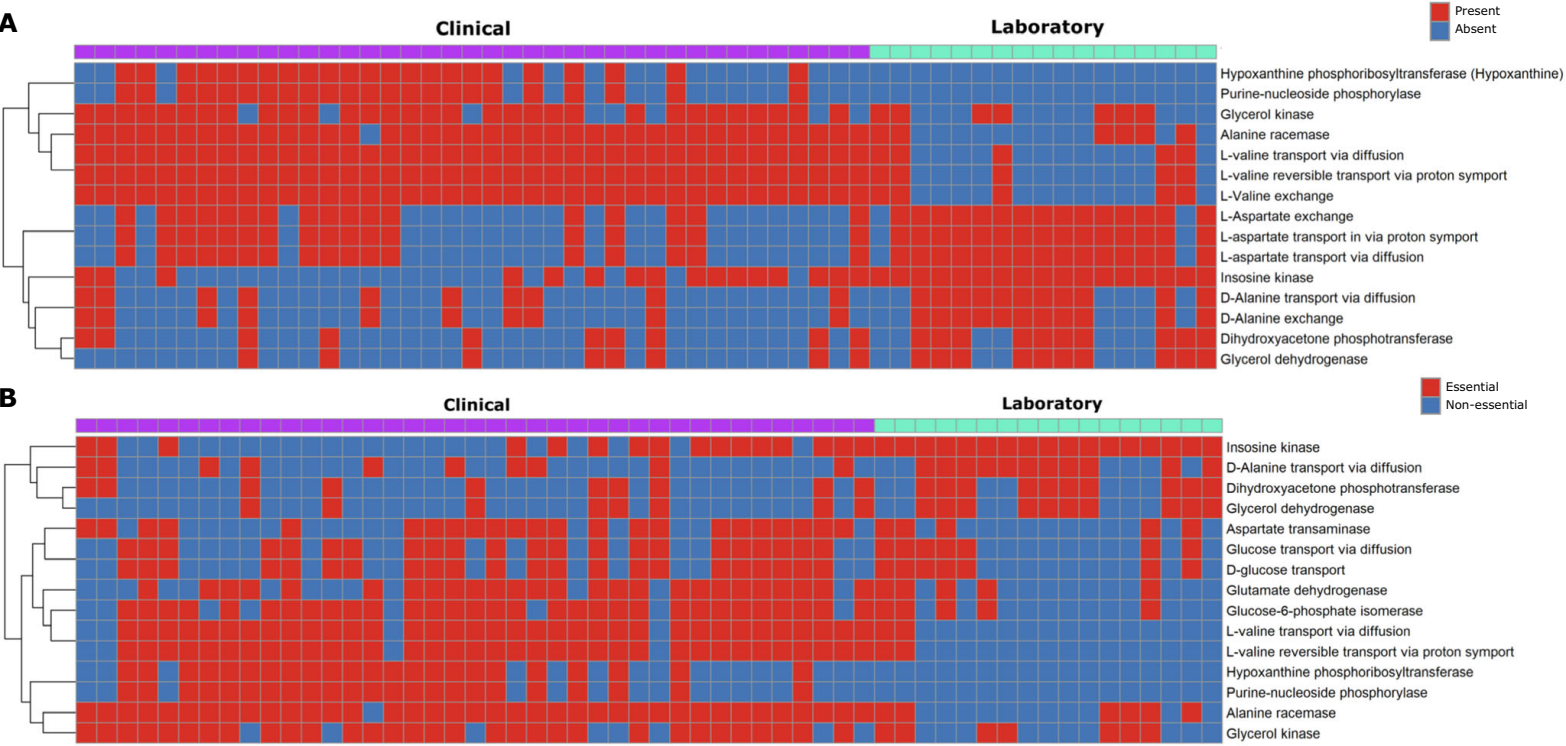
Environmental valine is differentially essential in clinical isolate context-specific models of *K. pneumoniae*. **A** Reaction topology differentially present between the context-specific model groups. **B** Differential reaction essentiality between isolate model groups, essentiality was determined through single reaction knockout screen with a cutoff of 1% of the biomass flux. Inclusion in final analysis was determined by cross reference against uncontextualized GENRE and a within-group shared threshold of >55% of models possessing a given feature. Color within the figure area indicates essentiality/presence (red) and non-essentiality/absence (blue), and color on the top margin denotes strain-type of origin for the associated transcriptome with clinical isolate (purple) or laboratory strain (teal).

To begin to assess overall differences in each contextualized model, we performed an analysis of unique subnetwork topology to each isolate-type-specific group of models. After subtracting “core” reactions that were present in all 56 models, we were left with a median of 52 and 72 reactions that were unique to either laboratory or clinical-specific models, respectively (Supplementary Table 3). Finally, to focus the analysis on those reactions most shared within each group we further limited the scope of reactions to only those shared by at least 55% of models within each group respectively, revealing 15 differentially active reactions (Fig. 3A). Among the most prominent patterns from this analysis were reactions for the import of environmental L-valine in clinical isolate models that were not present in their laboratory counterparts. When quantified, there was consistently a large positive net import flux across valine-associated reactions in clinical isolate models (mean net import flux = 8.959) (Supplementary Table 4). This finding was interesting as it has been recently discovered that exogenous L-valine promotes increased macrophage phagocytosis in vivo, thereby pressuring a lung pathogen to evolve to remove excess valine from the environment^23^.

Next, we sought to identify differentially essential metabolic pathway elements between clinical and laboratory isolates in an effort to ultimately provide a basis for future drug target discovery efforts. To accomplish this goal, we performed both single gene and reaction knockout simulations across context-specific models using a threshold of a minimum of 1% of optimal biomass for a gene or reaction to be deemed essential^38,39^. This functional analysis resulted in a median of 262 and 282 essential reactions in laboratory and clinical associated models, respectively (Supplementary Table 3). We then cross referenced these results against the uncontextualized iYL1228 to limit potential targets to only those components of metabolism that were environment-specific, and likely not due to strict user-applied constraints, as well as subtracting the “core” essential reactions, resulting in a reduction to a median 52 (laboratory) and 72 (clinical) essential reactions. Then, using a similar 55% threshold of shared elements to the previous topology analysis, our combined essentiality screen reported a total of 15 reactions as differentially essential between isolate types (Fig. 3B). This analysis indicated that bioconversion of environmental valine is essential in clinical isolates, but not for laboratory strains of *K. pneumoniae*. Of the 15 differentially essential reactions parsed, three reactions were directly related to valine metabolism (Fig. 3B). These three reactions had the highest levels of consistent essentiality among analyzed reactions, being essential for growth in ~90% of clinical isolate context-specific models. These results seem to agree with the prior topology-based findings, indicating that valine catabolism may play an important role in the metabolism of *K. pneumoniae* during infection.

### Simulated growth analysis predicts growth advantage and metabolic heterogeneity among clinical *K. pneumoniae* isolates

**Fig. 4:**
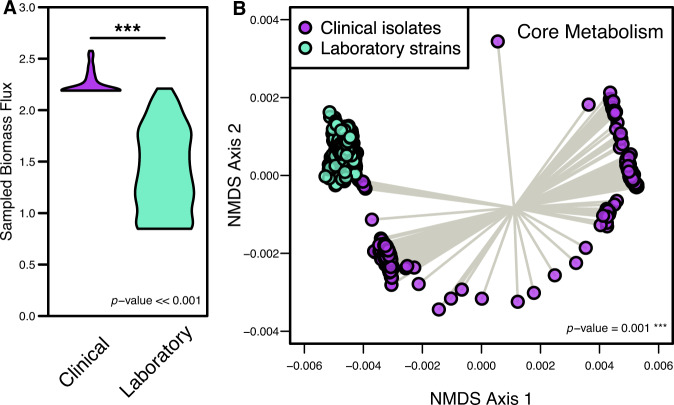
Growth rates and core metabolic activity significantly differ between clinical and laboratory isolate context-specific models. **A** Distribution of biomass synthesis reaction flux in each context-specific model, which relates to optimal growth rates. Significant difference determined by Wilcoxon rank-sum test. **B** Non-metric multidimensional scaling (NMDS) of Bray–Curtis dissimilarity between sampled context-specific flux distributions from infection and in vitro growth. Each point represents the collective activity of all reactions in a context-specific model during optimal growth conditions. Significant difference determined by PERMANOVA.

After observing consistent differences conserved across the metabolic models with individually integrated transcriptomes, we then performed a unified analysis within each group (clinical and laboratory) to incorporate all possible strain-level variation into single models of metabolism for each isolate type. We first specified the objective function as biomass for the subsequent analysis. We then iterated through a wide range of minimum objective flux values, assessing the correlation between the transcript abundances and the median reaction flux values during each iteration. From this analysis, the optimum biomass flux threshold value corresponding to the highest transcript-to-model correlation value was selected for both conditions. The two resultant models, being one laboratory strain model and one clinical isolate model, are able to account for the variation captured within each isolate type and the optimal flux distributions that most fit with the transcriptomic investments made by the bacterium. From the corresponding flux distributions, we first measured differences in sampled biomass reaction flux, which is analogous to the growth rate and accounts for biosynthesis of major cellular components (Fig. 4A). Strikingly, growth simulations with a rich medium in silico formulation predicted that the clinical isolate-specific model produced biomass at a significantly higher rate than laboratory strains under the same extracellular conditions (*p*-value ≪ 0.001), indicating that clinical isolates have a higher potential ability to grow more rapidly. This observation may be explained by the fact that colonizing a host organism presents substantial environmental pressure and encourages rapid growth to ensure the highest probability of colonization. Though it was observed that laboratory strains had potentially evolved metabolic machinery towards faster growth in culture medium, the growth simulations performed here demonstrated that clinical isolates may be able to grow more effectively due to their optimization around environmental pressures.

To then evaluate the degree to which core metabolic activity was altered across isolate types, we focused the analysis of reaction activity on reactions shared across both clinical and laboratory-specific models (excluding the biomass synthesis reaction). To accomplish this goal, flux sampling of the optimal metabolic solution space was performed exhaustively which resulted in possible activity levels for all reactions in the network that satisfies not only robust growth but also the integrated transcriptome. To focus specifically on differential patterns of core metabolism, we temporarily excluded biomass synthesis reaction components as they remained largely consistent across conditions. Using the reaction flux distributions from this subset of the overall metabolic networks, we performed unsupervised machine learning through non-metric multidimensional scaling (NMDS) of Bray–Curtis dissimilarities (Fig. 4B), which indeed showed a significant difference in core metabolic activity between the isolate-type-specific models (*p*-value = 0.001). This finding was intriguing as it indicated the strain groups are likely adapted for growth in distinct metabolic environments despite simulated growth in the same media conditions. Within-group variation was also significantly greater in clinical isolates (*p*-value < 0.001, Supplementary Table 5), accurately reflecting the environmental diversity within patients from which they were isolated. Conversely, the within-group variation of core metabolic activity was very low in laboratory-specific growth simulations, potentially suggestive of evolution toward growth in culture medium.

### Flux sampling further supports valine consumption as important to the metabolic strategy of clinical isolates

**Fig. 5:**
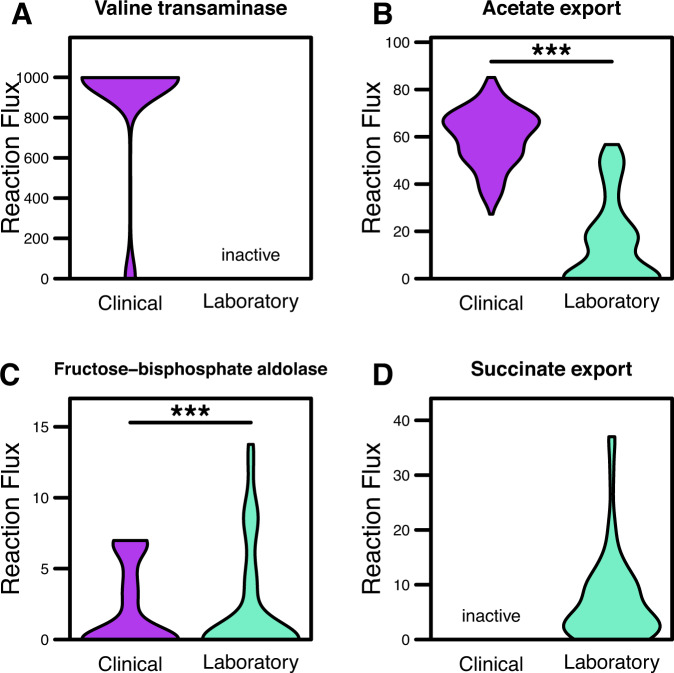
Sampled reaction flux in the metabolic models demonstrates ability of clinical isolates to optimize use of valine transaminase. **A** Valine transaminase (converts L-valine and pyruvate to 3-methyl-2-oxobutanoate and L-alanine), **B** acetate transport via diffusion, **C** fructose-biphosphate aldolase (converts D-fructose to D-glyceraldehyde 3-phosphate and dihydroxyacetone phosphate), or **D** succinate transport via proton symport. Inactive label indicates reactions only present in one context-specific model, and significant differences determined by Wilcoxon rank-sum test (***p-value ≤ 0.001).

Based on our previous results, we investigated differences in L-valine usage between the two isolate-type models during growth. Interestingly, the maximum growth rate achieved by the clinical-isolate model was significantly greater than that of the laboratory isolates (*p*-value ≪ 0.001). Closer investigation revealed the increased L-valine consumption was due to activity of valine transaminase, which mediates the conversion of L-valine to L-alanine and results in the downstream production and export of acetate (Fig. 5B). Valine transaminase was highly active only within the clinical isolate model, but entirely inactive from the laboratory strain-specific model following processing via Reaction Inclusion by Parsimony and Transcript Distribution (RIPTiDe) (Fig. 5B). RIPTiDe is a tool designed to integrate transcriptomic data into metabolic models and identify most likely forms of active metabolism by calculating the most cost-effective metabolic activity based on cellular investments into transcription. Differential transcription analysis for enzymes including in the valine biosynthesis KEGG pathway were indeed more highly transcribed in laboratory isolates (Supplementary Fig. 2A). Furthermore, although the gene for valine transaminase (*ilvE*, KPN_04269) is more highly transcribed in laboratory isolates, downstream enzymes in the valine degradation pathway are more highly transcribed in clinical isolates (Supplementary Fig. 2B). Thus, the use of RIPTiDe interestingly was able to reveal a likely reduction in valine consumption in laboratory isolates, and a larger rate of valine catabolism in clinical isolates. Alternatively, laboratory context-specific models were predicted to utilize D-fructose at significantly higher rates through the TCA cycle (Fig. 5C) and ultimately export large amounts of succinate as a byproduct (Fig. 5D), potentially due to laboratory media adaptation over time. These results support the hypothesis that the clinical isolates may be more primed to consume environmental valine, despite not being auxotrophic for the amino acid. As previously stated, exogenous L-valine has recently been shown to act in an immunostimulatory manner, causing an upregulation in macrophage phagocytosis in the host immune system^23^. Other studies have additionally shown that glutamate may play an immunosuppressive role, as accumulation of glutamate can lead to limited T-cell function^40^. Combined, these findings suggest clinical isolates of *K. pneumoniae* may have upregulated amino acid catabolism to combat host mechanisms of antibacterial immunity.

## Discussion

Throughout the past several years, alarmingly increasing numbers of bacterial pathogens have been reported as resistant to antibiotics^41^, emphasizing the need for identification of novel therapeutic options. GENREs have become powerful tools for elucidating the metabolic mechanisms underlying infectious diseases, allowing for the identification and acceleration of novel metabolism-based strategies for treatment^22^. Transcriptomic datasets have additionally been leveraged to further contextualize GENRES to discover essential portions of metabolism for the given cellular states^10^. By targeting elements of metabolism specifically related to life in a host, we may be able to interfere with the ability of the organism to colonize or cause disease. Here, we leveraged computational metabolic modeling of *K. pneumoniae* in combination with transcriptomic meta-analysis to identify unique components of clinical-isolate metabolism.

Metabolic modeling results indicated highly distinct patterns of activity within the core metabolism of clinical versus laboratory isolates, highlighting distinct adaptations to their individual environments. Analysis of the models representing clinical isolates point towards the conservation of valine metabolism machinery and the prioritization of early valine catabolism. Importantly, valine has been shown to augment macrophage phagocytosis, and this result could be indicative of an immunosuppressive strategy *K. pneumoniae* evolved for survival during infection^23^. Additional tracking of the pathways in which valine is metabolized showed that clinical isolates were converting this amino acid into glutamate, which is thought to act as an immunosuppressant. This phenotype may be due to *K. pneumoniae* evolving to sequester valine from the host immune system^23^. The bacteria are then able to convert and excrete the byproducts as glutamate which acts as an immunosuppressant signal. This observation agrees with other studies that have shown the ability to metabolize valine has a clear effect on the fitness of *K. pneumoniae* during active infection^42,43^. Cumulatively, this study points towards the importance of amino acid catabolism for successful host colonization, a functionality that may be conserved among strains more recently isolated from infections.

While this study presents several novel insights into the relationship between the metabolism of *K. pneumoniae* and host factors, some limitations to the analyses are present. While transcriptomic surveys have become relatively standard, there are still potential issues including technical variability and sample heterogeneity which may influence the quality of data in each study^11,12^. Additionally, since the strict exclusion criteria used for selecting datasets resulted in a fewer number of studies being included in this analysis, none of which included both laboratory strains and clinical isolates in the same study, there could be some bias introduced due to this data selection. Further, 15 of the 17 laboratory strains analyzed within this study originated from the same parent strain, which may have introduced strain bias into this study. Considering these factors, a transcriptomic meta-analysis addresses some of the limiting factors in each component study. We additionally acknowledge that GENREs are not a complete representation for all mechanisms that determine metabolic activity, as they are only built around current reaction annotation data and lack consideration for other levels of regulation^44^. Despite this limitation, the GENRE utilized here was able to accurately predict the metabolic capabilities of *K. pneumoniae* previously^37^, bolstering confidence in the metabolic predictions made here. We do understand that since the selected model (iYL1228) was curated using *K. pneumoniae* strain MGH 78578, the diversity of strains the transcriptomic datasets were gathered from may result in a differing ability for the selected model to fully represent the metabolic activity of each of these strains. Furthermore, while the discordant relationship with higher valine transaminase transcription in laboratory strains yet lower reaction activity is most likely due to increased transcription of functionally related enzymes in clinical isolates, inaccuracies in construction of the GENRE may also be a contributing factor that requires additional curation. Despite these considerations, our analyses demonstrate the strength of systems biology approaches to identify potential metabolic targets against bacterial pathogens.

Our results indicate that increased valine catabolism is a metabolic phenotype more closely associated with clinical isolates of *K. pneumoniae*. Future studies may build on the targets identified in this study to investigate the role of L-valine in *K. pneumoniae* colonization and virulence or amino acid release and utilization by immune cells. Finally, the methods described here may be applied to other recalcitrant bacterial pathogens in the future as a platform for accelerated drug target discovery.

## Methods

### Transcriptomic data and read curation

All transcriptomic datasets were obtained from the Sequence Read Archive (SRA) in FASTQ format using the SRA Toolkit. Raw reads were quality trimmed using Sickle^45^ to ≥Q25, a tool that uses quality and length thresholds to determine the appropriate location to trim the 5’- and 3’- end of reads (Supplementary Table 6). These reads were then strictly mapped to the *K. pneumoniae* MGH 78578 genes (GenBank accession number: CP000647.1) using Bowtie2^46^, a tool for aligning sequencing reads to a designated reference sequence, and screened for optical/PCR duplicate reads with Picard MarkDuplicates^47^, an algorithm for locating and tagging duplicate reads originating from a single fragment of DNA. Mapping files were converted to human-readable format using SAMtools^48^, a tool designed to facilitate alignment manipulation in the common Sequence Alignment/Map (SAM) format. Transcript abundances were normalized to both read and target gene lengths then evenly subsampled for equal comparison across conditions.

### GENRE-based analyses

The GENRE of *Klebsiella pneumoniae* strain MGH 78578, iYL1228^37^, was obtained from the BiGG Model database^49^ on 5/21/20. Flux analyses performed in this study utilized cobrapy (v0.22.1)^50^. Growth simulations were performed using a previously published rich medium in silico formulation^51^. Gene and reaction essentiality screens were both performed with a minimum objective flux threshold of 1.0% of the optimal value, the commonly accepted threshold for these simulations^39^. Replicate GENRE transcriptome integration was performed with RIPTiDe (v3.2.3)^36^ with 0.75 minimum objective flux fraction. RIPTiDe is a tool developed to use both transcriptomic abundances and overall flux parsimony to identify the most cost-effective usage of metabolism while still accurately reflecting the inputted transcriptomic data. To prune the model so that it represents the most focused and biologically feasible metabolic solution space possible, RIPTiDe first sets the objective function to carry near optimal flux. Transcriptomic abundance values are then used to assign linear coefficients to each reaction, prior to performing an optimization of the minimum sum of fluxes. Flux balance analysis is then used to identify the reactions that no longer carry flux, and these reactions are subsequently pruned from the model. Maximum fit RIPTiDe analysis, where all minimum objective flux fractions are iteratively tested to return the model with the best correlation between the context-specific flux reactions and the inputted transcriptomic values, was performed with all transcriptome replicates on the default settings.

### Statistical analysis

Statistical analyses were performed in R (v3.2.0). Ordination analysis was accomplished using the vegan package (v2.5.7)^52^.

## Supplementary information

Supplemental Material

Supplementary Table 1

Supplementary Table 2

Supplementary Table 3

Supplementary Table 4

Supplementary Table 5

Supplementary Table 6

## Data Availability

All data used and generated in this study are available in the GitHub repository associated with this study (https://github.com/mjenior/Klebsiella_2021).

## Code availability

Complete analysis associated with this study is available in a Jupyter Notebook hosted in a GitHub repository along with all generated models and code associated with this study (https://github.com/mjenior/Klebsiella_2021).
