# Supplementary material for: Genome-scale metabolic modeling reveals increased reliance on valine catabolism in clinical isolates of Klebsiella pneumoniae
Source: NPJ Syst Biol Appl. 2022 Oct 28;8:41. doi: 10.1038/s41540-022-00252-7 (PMC9616910; doi:10.1038/s41540-022-00252-7)
Supplement: Supplementary file 1 — Supplemental Material [file 41540_2022_252_MOESM1_ESM.pdf]

Supplementary Materials

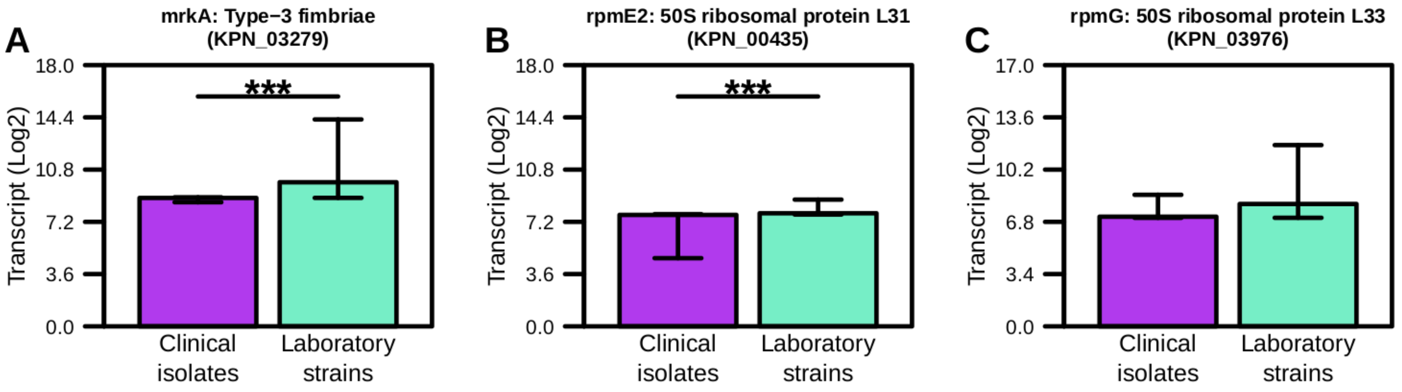

Supplementary Figure 1) Additional differentially expressed genes from Figure 1. Median and interquartile ranges from pooled analysis between clinical and laboratory isolate transcriptomes. Significant differences determined by Wilcoxon rank-sum test with Benjamini-Hochberg correction (*** *p*-value ≤ 0.001).

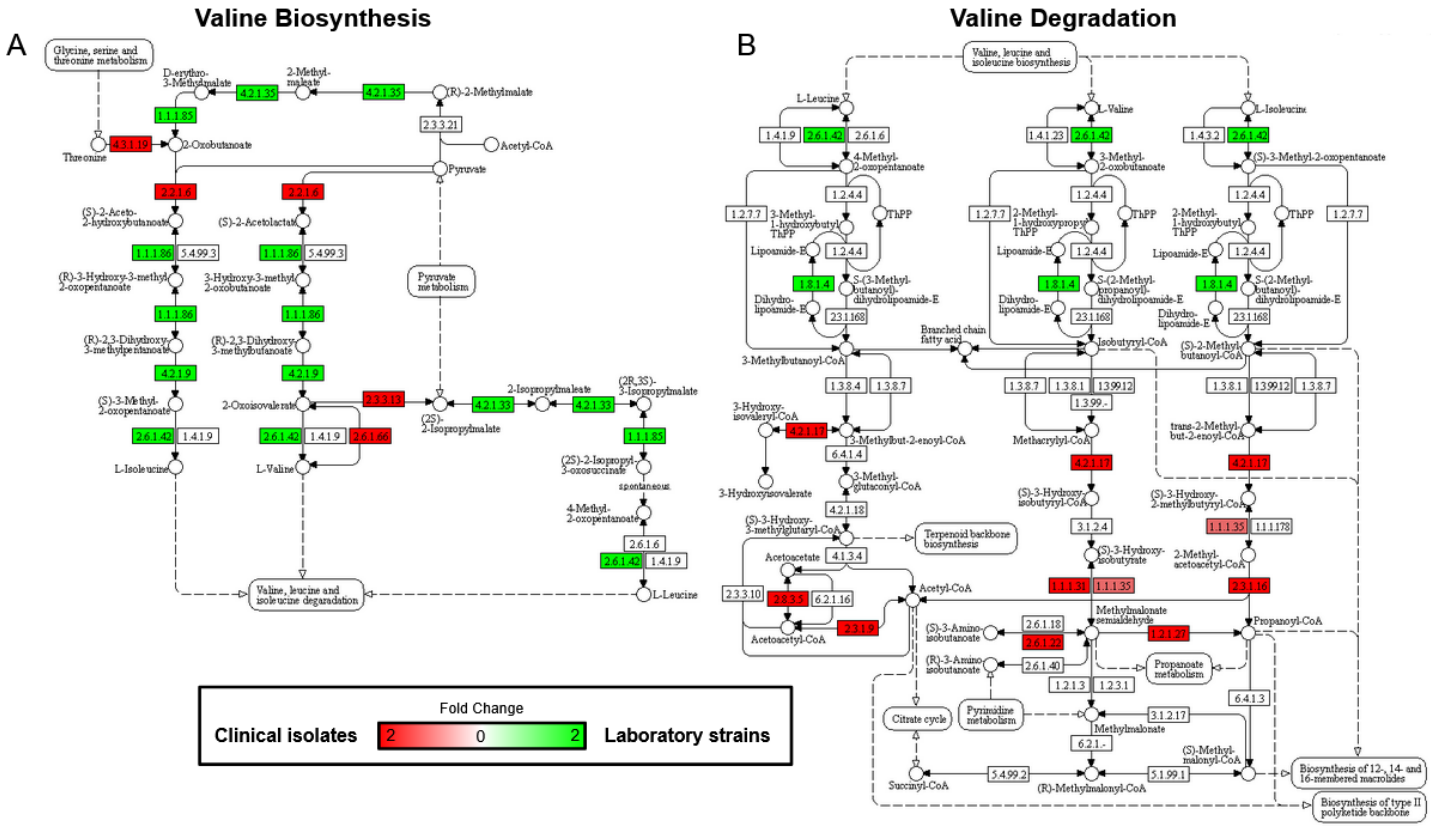

Supplementary Figure 2 | Differential expression of valine biosynthesis and degradation pathways. Fold change differential expression analysis across all analyzed transcriptomes in pathways associated with both

KEGG Valine **(A)** Biosynthesis or **(B)** Degradation. Red represents areas of the pathway that are more highly transcribed in clinical isolates, and green represents areas that are more highly transcribed in laboratory strains.

Supplementary Table 1 | *K. pneumoniae* transcriptomic dataset metadata

Supplementary Table 2 | Differential expression analysis summary statistics

Supplementary Table 3 | Complete gene and reaction essentiality results

Supplementary Table 4 | Valine-associated reaction net import flux quantification

Supplementary Table 5 | Flux analysis summary table

Supplementary Table 6 | RNA-Seq mapping summary table
